# A atenção à saúde da mulher no Estado de São Paulo, Brasil: avaliação de serviços de atenção primaria à saúde

**DOI:** 10.1590/0102-311XPT147024

**Published:** 2025-06-27

**Authors:** Caroline Eliane Couto, Luceime Olívia Nemes, Thais Fernanda Tortorelli Zarili, Carolina Siqueira Mendonça, Eduardo de Sousa Gomes, Stella Bianca Gonçalves Brasil Pissatto, Hélio Rubens de Carvalho Nunes, Elen Rose Lodeiro Castanheira

**Affiliations:** 1 Faculdade de Medicina Botucatu, Universidade Estadual Paulista Júlio de Mesquita Filho, Botucatu, Brasil.; 2 Centro Universitário UniFaveni, Guarulhos, Brasil.

**Keywords:** Saúde da Mulher, Atenção Primária à Saúde, Avaliação em Saúde, Qualidade da Assistência à Saúde, Women's Health, Primary Health Care, Health Evaluation, Quality of Health Care, Salud de la Mujer, Atención Primaria de Salud, Evaluación en Salud, Calidad de la Atención de Salud

## Abstract

O objetivo deste trabalho é avaliar a organização da atenção à saúde da mulher em serviços de atenção primária à saúde. Trata-se de pesquisa avaliativa que utilizou indicadores extraídos de inquérito realizado no Estado de São Paulo, Brasil, em 2022 pelo sistema QualiAB. Foram avaliados 2.269 serviços, localizados em 356 municípios por meio de uma matriz avaliativa com 46 indicadores reunidos em cinco domínios. Calculou-se o percentual de desempenho a partir da média geral obtida. Com uso da técnica de K-médias os serviços foram agrupados em três *clusters* de qualidade, analisando-se as associações destes com características dos serviços por meio de regressão linear com resposta multinomial. O desempenho geral dos 2.269 serviços avaliados foi 66,4%. O maior desempenho foi do domínio “atenção ao puerpério” (81,6%), seguido por “atenção ao planejamento reprodutivo” (68,8%), “prevenção e rastreamento dos cânceres de mama e útero” (68,6%), “atenção ao pré-natal” (61,9%) e “atenção às mulheres em situação de violência” (47,2%). Observou-se maior frequência de ações “tradicionais”, relativas ao eixo materno-infantil e menor frequência de ações de educação e promoção à saúde. As variáveis “porte populacional” (> 200 mil habitantes), “captação de gestantes por agentes comunitários”, “utilização de dados epidemiológicos” e “periodicidade das reuniões de equipe” tiveram associação positiva com a maior qualidade. As fragilidades identificadas estão sob governabilidade de equipes locais e da gestão municipal e apontam a necessidade de qualificação profissional e reorganização dos processos de trabalho para efetivação das diretrizes para a atenção integral à saúde da mulher.

## Abstract (en)

This study aimed to assess the organization of women’s health care within primary health care services. This evaluative research utilized indicators derived from a survey conducted in the state of São Paulo, Brazil, in 2022 by the QualiAB system. A total of 2,269 services, located in 356 municipalities, were evaluated using an assessment matrix comprising 46 indicators grouped into five domains. Overall performance was estimated based on the average score obtained. Using the K-means clustering technique, the services were grouped into three quality clusters, and associations between these clusters and service characteristics were analyzed using multinomial linear regression. The overall performance of the 2,269 evaluated services was 66.4%. The highest performance was observed in the “postpartum care” domain (81.6%), followed by “reproductive planning” (68.8%), “breast and cervical cancer prevention and screening” (68.6%), “prenatal care” (61.9%), and “care for women in situations of violence” (47.2%). A higher frequency of “traditional” actions related to maternal and child health was observed, along with a lower frequency of health education and promotion activities. The variables “population size (> 200,000 inhabitants)”, “recruitment of pregnant women by community agents”, “use of epidemiological data”, and “frequency of team meetings” were positively associated with higher quality. The identified weaknesses lie within the governance of local teams and municipal management, highlighting the need for professional development and the reorganization of work processes to effectively implement the guidelines for comprehensive women’s health care.

## Resumen

El objetivo de este estudio es evaluar la organización de la atención a la salud de la mujer en los servicios de atención primaria de salud. Se trata de una investigación evaluativa que utilizó indicadores de una encuesta realizada en el estado de São Paulo, Brasil, en 2022 por el sistema QualiAB. Se evaluaron un total de 2.269 servicios ubicados en 356 municipios mediante una matriz de evaluación con 46 indicadores reunidos en cinco dominios. El porcentaje de rendimiento se calculó a partir del promedio general obtenido. A partir del uso de la técnica K-medias, los servicios se agruparon en tres clústeres de calidad, en el cual se analizó sus asociaciones con las características del servicio mediante la regresión lineal con respuesta multinomial. El rendimiento global de los 2.269 servicios evaluados fue del 66,4%. El mayor desempeño fue en el dominio “atención al puerperio” (81,6%), seguido de “atención a la planificación reproductiva” (68,8%), “prevención y cribado de cánceres de mama y útero” (68,6%), “atención al prenatal” (61,9%) y “atención a mujeres en situación de violencia” (47,2%). Hubo una mayor frecuencia de acciones “tradicionales” relacionadas con el eje madre-hijo y una menor frecuencia de acciones de educación y promoción de la salud. Las variables “tamaño poblacional” (> 200.000 habitantes), “reclutamiento de mujeres embarazadas por agentes comunitarios”, “uso de datos epidemiológicos” y “frecuencia de reuniones de equipo” se asociaron positivamente con mayor calidad. Las debilidades identificadas están bajo la gobernabilidad de los equipos locales y de la gestión municipal y apuntan a la necesidad de una capacitación profesional y reorganización de los procesos de trabajo para implementar los lineamientos dirigidos a la atención integral a la salud de las mujeres.

## Introdução

A atenção à saúde da mulher no Brasil vivenciou um importante avanço com a criação da Política Nacional de Atenção Integral à Saúde da Mulher (PNAISM) em 2004, trazendo para a atenção primária à saúde (APS) um papel fundamental nesse processo. É neste nível de atenção em que são realizadas a grande maioria das ações de atenção à saúde das mulheres, principalmente no âmbito da promoção à saúde e prevenção de doenças, por meio de ações educativas, atenção ginecológica, atenção ao pré-natal e puerpério, rastreamento do câncer de mama e colo de útero, planejamento reprodutivo, entre outros ^1^ ^,^ ^2^.

A expansão de serviços de APS desde a década de 1990 ampliou o acesso das mulheres ao cuidado em saúde. Entretanto, estudos apontam a persistência de importantes desafios para a efetivação do cuidado integral proposto pela PNAISM na APS ^3^ ^,^ ^4^, tais como atenção inadequada ao pré-natal ^5^ ^,^ ^6^ ^,^ ^7^, baixa cobertura de rastreamento de câncer de colo de útero ^8^ ^,^ ^9^, além das desigualdades que permeiam o acesso aos serviços de saúde ^2^ ^,^ ^3^ ^,^ ^10^.

Estudo desenvolvido utilizando dados do segundo ciclo do Programa de Melhoria do Acesso e da Qualidade (PMAQ) em 2014 relata que a operacionalização da assistência às mulheres no Brasil ainda está distante do preconizado pela PNAISM, com grandes diferenças entre as macrorregiões do país. A Região Sudeste mostrou melhor classificação em quase todas os grupos temáticos, exceto no “desenvolvimento das ações” ^11^. Em consonância, Schönholzer et al. ^12^ trazem que, apesar do aumento significativo nas taxas dos indicadores do Previne Brasil relacionados à saúde das mulheres entre 2020 e 2021, poucos estados brasileiros alcançaram as metas estipuladas nacionalmente para esses indicadores, ficando em destaque novamente a Região Sudeste pelo melhor desempenho.

São Paulo é o estado mais populoso do Brasil e apresenta uma enorme pluralidade de arranjos geográficos, sociais e culturais, que impacta diretamente na forma como a Rede de Atenção à Saúde e, consequentemente, os serviços de APS são organizados, ofertados e acessados ^13^ ^,^ ^14^. Nesse sentido, olhar para a organização do cuidado da atenção à saúde da mulher nos serviços de APS do Estado de São Paulo permite identificar variáveis do processo de trabalho que indicam o alcance dos resultados esperados, conforme proposto pelo modelo donabediano ^15^. É importante avaliar o quanto as práticas desenvolvidas nos serviços se aproximam das recomendações trazidas pela PNAISM e protocolos direcionados à saúde da mulher, buscando compreender quais pontos devem ser fortalecidos na busca da efetivação de uma assistência à saúde integral e de qualidade para as mulheres.

No Brasil, a avaliação é uma ferramenta ainda subutilizada no cotidiano dos gestores ^16^ ^,^ ^17^ ^,^ ^18^, contudo, sua utilização pode implicar melhorias significativas nos processos de trabalho e em seus resultados. Mesmo que a avaliação não gere medidas imediatas, espera-se que contribua para um julgamento que influencie positivamente nas decisões dos gestores e das equipes de saúde ^16^ ^,^ ^17^ ^,^ ^18^.

Considerando o exposto, este estudo tem como objetivo avaliar a organização da atenção à saúde da mulher em serviços de APS no Estado de São Paulo.

## Método

Trata-se de uma pesquisa avaliativa, de corte transversal, com foco na organização do processo de trabalho de serviços de APS na atenção à saúde da mulher, para qual utilizou-se dados secundários, provenientes do inquérito realizado no Estado de São Paulo com o *Sistema de Avaliação e Monitoramento de Serviços de Atenção Básica* (QualiAB), em 2022.

O QualiAB é um instrumento de avaliação da qualidade dos serviços de APS, originalmente desenvolvido por pesquisa avaliativa realizada em 2007, sendo atualizado e revalidado nacionalmente em 2015 ^19^ ^,^ ^20^. Refere-se a um questionário fechado, autoaplicado eletronicamente e direcionado aos gerentes e equipes de serviços de APS, tendo como foco a organização das práticas dos serviços, contendo indicadores de estrutura e processo ^15^ do conjunto diversificado de ações sob responsabilidade dos serviços de APS ^20^. A participação no inquérito requer adesão voluntária dos municípios e, em seguida, dos serviços de APS.

No ano de 2022 foi disponibilizada a participação no inquérito QualiAB a todos os 645 municípios do Estado de São Paulo. Aderiram ao inquérito 356 municípios paulistas, com 2.269 serviços de APS com respostas completas ao questionário, compondo o universo de serviços a serem avaliados nesse estudo.

Para avaliação da qualidade na organização das ações de atenção à saúde da mulher na APS, foi desenvolvida uma matriz de análise e julgamento a partir de pesquisa documental e bibliográfica pertinente ao tema, e que foi utilizada para selecionar as variáveis presentes no inquérito de 2022. Os principais documentos norteadores utilizados foram: *Política Nacional de Atenção Integral à Saúde da Mulher: Princípios e Diretrizes* ^1^; *Protocolo da Atenção Básica: Saúde das Mulheres* ^21^; *Protocolo Clínico e Diretrizes Terapêuticas para Prevenção da Transmissão Vertical de HIV, Sífilis e Hepatites Virais* ^22^; as normas e manuais técnicos da série *Cadernos de Atenção Básica* (nº 26 - *Saúde Sexual e Saúde Reprodutiva* ^23^; e nº 32 - *Atenção ao Pré-natal de Baixo Risco* ^24^). Também foi realizada uma revisão da literatura com busca nas bases de dados PubMed e LILACS, utilizando os descritores “saúde da mulher”, “qualidade da assistência” e “atenção primária à saúde”, com levantamento de estudos relacionados à temática.

A matriz avaliativa referente à dimensão “atenção à saúde da mulher” é composta por 46 variáveis extraídas do questionário do QualiAB, que foram consideradas como indicadores da qualidade da organização do processo de trabalho dos serviços, e reunidas em cinco domínios de análise: atenção ao planejamento reprodutivo; atenção às mulheres em situação de violência; prevenção e rastreamento dos cânceres de mama e útero; atenção ao pré-natal de baixo risco; e atenção ao puerpério.

A extração de informações do banco original no inquérito QualiAB permitiu a construção de uma base de dados composta pelos indicadores da matriz avaliativa. As respostas dos serviços a esses indicadores foram dicotomicamente categorizadas, no qual 1 correspondia ao que o serviço referia fazer; e 0 ao que o serviço não referia fazer.

A fim de entender melhor o perfil do conjunto de serviços avaliado, foram utilizadas as seguintes variáveis de caracterização: tipo de serviço (unidade de saúde da família - USF; unidade básica de saúde “tradicional” - UBS; ou unidades “mistas” - UBS/USF), localização (rural ou urbana), instituição que exerce gestão administrativa (Secretária Municipal de Saúde; organização ou fundação social; e outros), periodicidade de reunião de equipe (semanal ou quinzenal; mensal; bimestral ou intervalos maiores; e não realizar reunião), utilização de dados epidemiológicos para planejamento da unidade, presença de agente comunitário de saúde (ACS) na equipe e se a unidade faz captação de gestantes pelos ACS. As UBS com componentes de saúde da família foram consideradas como unidades “mistas”: UBS com Programa de Agentes Comunitários de Saúde (PACS) ou Estratégia Saúde da Família (ESF).

Inicialmente foram calculadas as frequências relativas e absolutas dos serviços com respostas positivas para cada indicador da matriz avaliativa e para as variáveis de caracterização dos serviços. Posteriormente, foi realizado o cálculo do desempenho por serviço para cada domínio e na dimensão avaliativa de “atenção à saúde da mulher”. Com base na distribuição das médias de desempenho por serviço, foi calculado o desempenho médio do conjunto de serviços de APS avaliados, por domínio e na dimensão avaliativa.

A partir do desempenho dos serviços nos cinco domínios, estes foram agrupados com uso da técnica K-médias com o objetivo de diferenciar grupos de qualidade. A opção pela divisão em três *clusters* se mostrou adequada para análise, uma vez que cada grupo apresentou boa distribuição interna e distanciamento entre os grupos.

Com o objetivo de investigar fatores associados à qualidade dos serviços, os grupos foram associados a variáveis independentes por meio do ajuste de regressão linear com resposta multinomial. Desse modo, os três *clusters* ou grupos de qualidade (G3 - maior qualidade; G2 - intermediária; G1 - menor qualidade) representam a variável dependente, tratados como níveis de uma variável categórica. As variáveis independentes utilizadas foram as variáveis de caracterização descritas anteriormente e o porte populacional dos municípios, que foram classificados em quatro estratos: < 10 mil habitantes; entre 10 mil e 50 mil habitantes; entre 50 mil e 200 mil habitantes; e > 200 mil habitantes. Para o ajuste dos modelos, o grupo G1 foi adotado como sendo o grupo de referência. As associações foram consideradas estatisticamente significativas se p < 0,05. As análises foram realizadas com o software SPSS 21 (https://www.ibm.com/).

Este estudo foi aprovado pelo Comitê de Ética em Pesquisa da Faculdade de Medicina de Botucatu da Universidade Estadual Paulista Júlio de Mesquita Filho (parecer nº 6.530.130, aprovado em 24 de novembro de 2023).

## Resultados 

Foram analisados 2.269 serviços de APS com a seguinte caracterização: USF (49,4%), seguidos por unidades “mistas” (31,7%) e UBS (18,9%); sendo a localização principal urbano-periférica (55,9%); e apresentando, preponderante, o município como a instituição responsável pela gestão administrativa (87,6%) (Tabela 1).

**Tabela 1 Caracterização:** dos serviços participantes do *Sistema de Avaliação e Monitoramento de Serviços de Atenção Básica* (QualiAB) no Estado de São Paulo, Brasil, 2022.

Variável de caracterização dos serviços	n (N = 2.269)	%
Estrato populacional (habitantes)		
< 10.000	256	11,3
10.000-50.000	678	29,9
50.000-200.000	718	31,6
> 200.000	617	27,2
Tipo de serviço de atenção primária		
USF	1.122	49,4
UBS tradicional	428	18,9
Mista	719	31,7
Instituição que assume gestão administrativa		
Secretaria/Coordenação/Diretoria Municipal de Saúde	1.987	87,6
Fundação ou Organização Social	237	10,4
Outro	45	2,0
Localização geográfica		
Rural	204	9,0
Urbano-central	796	35,1
Urbano-periférica	1.269	55,9
Presença de ACS na equipe		
Não	416	18,3
Sim	1.853	81,7
Captação de gestantes por ACS		
Não	528	23,3
Sim	1.741	76,7
Utilização de dados epidemiológicos da população adscrita no planejamento		
Não	555	24,5
Sim	1.714	75,5
Periodicidade de reunião de equipe		
Semanal	828	36,5
Quinzenal	286	12,6
Mensal	640	28,2
Bimestral ou intervalos maiores	160	7,1
Apenas discussões de caso	279	12,3
Não ocorrem reuniões	76	3,3

ACS: agente comunitário de saúde; UBS: unidade básica de saúde; USF: unidade de saúde da família.

São apresentados também na Tabela 1 dados como a periodicidade de reunião de equipe nas unidades de saúde, sendo as mais frequentes as reuniões semanais (36,5%) e mensais (28,2%). A maior parte das unidades (75,5%) referiu a utilização de dados epidemiológicos em seu planejamento. Das 2.269 unidades participantes, a maioria tinha ACS em sua equipe (81,7%) e referiu a realização de captação de gestante por esses profissionais (76,7%).

A frequência obtida para os indicadores de cada domínio da dimensão avaliativa “atenção à saúde da mulher” é apresentada na Tabela 2. Foi observada maior frequência dos indicadores referentes a ações do eixo materno-infantil, realizadas na atenção ao pré-natal e puerpério, como a realização do teste de gravidez na urina (89,7%); convocação de gestantes faltosas (93,6%); realização de tratamento para sífilis na gestante e parceiro (89,5%); assim como orientações sobre aleitamento materno no puerpério imediato e/ou tardio (95,5%), orientações sobre o atendimento do recém-nascido (90,5%) e avaliação de condições de nascimento (89,6%).

**Tabela 2 Frequência:** absoluta e relativa dos indicadores da matriz avaliativa de atenção à saúde da mulher, por domínios. *Sistema de Avaliação e Monitoramento de Serviços de Atenção Básica* (QualiAB), Estado de São Paulo, Brasil, 2022.

Indicador	n (N = 2.269)	%
Domínio: Atenção ao planejamento reprodutivo		
Ações educativas sobre planejamento reprodutivo realizadas na comunidade	986	43,5
Ações educativas sobre gravidez na adolescência voltadas para adolescentes	1.366	60,2
Ações de planejamento reprodutivo na atenção aos adolescentes	1.238	54,6
Disponibilidade do anticoncepcional oral	1.696	74,7
Disponibilidade do anticoncepcional injetável	1.826	80,5
Disponibilidade do preservativo masculino	2.238	98,6
Disponibilidade do preservativo feminino	1.998	88,1
Disponibilidade do dispositivo intrauterino (DIU)	1.137	50,1
Disponibilidade da pílula do dia seguinte (contracepção de emergência)	1.056	46,5
Disponibilidade do encaminhamento para laqueadura	2.071	91,3
Domínio: Atenção às mulheres em situação de violência		
Ações educativas sobre violência realizadas na comunidade	728	32,1
Ações educativas sobre violência realizadas na unidade	790	34,8
Encaminhamento para serviço de referência e acompanhamento na unidade nos casos de violência contra a mulher	1.095	48,3
Utilização de protocolo de atendimento como estratégia para detecção da violência contra a mulher	967	42,6
Utilização de discussão de caso em equipe como estratégia para detecção da violência contra a mulher	1.261	55,6
Sensibilização e capacitação da equipe para detecção da violência contra a mulher	1.154	50,9
Notificação compulsória nos casos de detecção de violência contra a mulher	1.503	66,2
Domínio: Prevenção e rastreamento dos cânceres de mama e útero		
Ações educativas sobre prevenção do câncer de colo de útero e mama realizadas na unidade	1.941	85,5
Coleta de Papanicolau duas ou mais vezes ao ano para mulheres com resultado anterior alterado	1.609	70,9
Adequação dos critérios de coleta de Papanicolau para população em idade alvo de rastreamento, segundo protocolos vigentes	1.022	45,0
Realização de exame físico sempre que há queixas da paciente em relação às mamas	2.190	96,5
Discussões em fóruns na comunidade e campanhas sobre câncer de mama	720	31,7
Adequação dos critérios de solicitação de mamografia para população com risco elevado de câncer de mama, segundo protocolos vigentes	1.934	85,2
Adequação dos critérios de solicitação de mamografia para população em idade alvo de rastreamento, segundo protocolos vigentes	1.477	65,1
Domínio: Atenção ao pré-natal de baixo risco		
Realização do teste de gravidez na urina	2.035	89,7
Ao realizar teste de gravidez, considera-se se a gravidez é desejada ou não antes de dar o resultado e fazer os encaminhamentos necessários	40	1,8
Realização do primeiro atendimento por profissional da enfermagem imediatamente após diagnóstico da gravidez	1.886	83,1
Convocação de gestantes faltosas em atividades e consultas agendadas	2.124	93,6
Solicitação de sorologia/teste rápido de sífilis no primeiro e terceiro trimestre gestacional	1.732	76,3
Solicitação de sorologia/teste rápido de HIV no primeiro e terceiro trimestre gestacional	1.707	75,2
Solicitação dos exames de rotina para todas as gestantes (exceto sorologia sífilis e HIV)	1.986	87,5
Realização de grupos educativos para gestantes	1.538	67,8
Organização de visita prévia da gestante e de seu parceiro à maternidade	710	31,3
Encaminhamento da gestante de alto risco para serviço de referência mantendo o acompanhamento na unidade	1.562	68,8
Realização de tratamento de sífilis com penicilina benzatina na unidade para a gestante e parceiro	2.030	89,5
Realização de atendimento diferenciado para gestantes de 10 a 19 anos	901	39,7
Domínio: Atenção ao puerpério		
Ações educativas sobre aleitamento materno realizadas na unidade	1.628	71,7
Convocação de faltosos em atividades e consultas agendadas de puerpério/Revisão pós-parto	1.715	75,6
Avaliação das condições de nascimento do recém-nascido e orientação sobre cuidados básicos no puerpério imediato	2.032	89,6
Orientação para o atendimento de rotina do recém-nascido (vacinação, exames, outros) no puerpério imediato	2.053	90,5
Orientação sobre atividade sexual e contracepção no puerpério imediato	1.950	85,9
Avaliação de sinais de sofrimento mental relacionado ao puerpério	1.762	77,7
Orientação sobre planejamento reprodutivo e contracepção durante o aleitamento no puerpério tardio	2.005	88,4
Orientação sobre aleitamento materno no puerpério	2.165	95,4
Avaliação das condições sociofamiliares (rede de apoio, trabalho) no puerpério	1.714	75,5
Orientação sobre direitos reprodutivos, sociais e trabalhistas no puerpério	1.483	65,4

Também é observada a alta frequência dos indicadores de disponibilidade de métodos anticoncepcionais nos serviços, principalmente preservativos masculino (98,8%) e feminino (88,8%), encaminhamento para laqueadura (91,3%) e anticoncepcional injetável (80,5%). No domínio “prevenção e rastreamento dos cânceres de mama e útero” observou-se uma maior frequência de indicadores relacionados à realização de exame físico quando há queixas das pacientes (96,5%) e adequação dos critérios de solicitação de exames de rastreamento para mamografia em população com elevado risco de câncer (85,2%).

Os indicadores que obtiveram menores frequências foram relacionados a ações de promoção à saúde e ações educativas, com exceção do indicador de ações educativas relacionadas à prevenção do câncer de colo de útero e mama (85,5%). Ações educativas sobre planejamento reprodutivo realizadas na comunidade (43,5%), assim como ações educativas sobre violência realizadas na comunidade (32,1%) e na unidade (34,8%) foram alguns dos indicadores com menores frequências nos serviços. O indicador “ao realizar teste de gravidez, considera se a gravidez é desejada ou não antes de dar o resultado e fazer os encaminhamentos necessários” teve a menor frequência dentre os serviços avaliados (1,8%).

O conjunto de serviços de APS paulistas participantes obteve desempenho de 66,4% na dimensão avaliativa “atenção à saúde da mulher”, conforme Tabela 3. O domínio com maior desempenho foi o de “atenção ao puerpério” (81,6%), seguido do “atenção ao planejamento reprodutivo” (68,8%), “prevenção e rastreamento dos cânceres de mama e útero” (68,6%) e “atenção ao pré-natal de baixo risco” (61,9%). O domínio com menor desempenho foi o de “atenção às mulheres em situação de violência” (47,2%).

**Tabela 3 Desempenho:** do conjunto de serviços, por domínios e dimensão avaliativa de atenção à saúde da mulher, segundo medidas de tendência central. *Sistema de Avaliação e Monitoramento de Serviços de Atenção Básica* (QualiAB), Estado de São Paulo, Brasil, 2022.

Domínio	Média	Mediana	DP	Mínimo	Máximo
Atenção ao planejamento reprodutivo	68,8	70,0	20,7	0,0	100,0
Atenção às mulheres em situação de violência	47,2	42,9	30,7	0,0	100,0
Prevenção e rastreamento dos cânceres de mama e útero	68,6	71,4	21,5	0,0	100,0
Atenção ao pré-natal de baixo risco	61,9	61,5	15,8	0,0	92,3
Atenção ao puerpério	81,6	90,0	23,1	0,0	100,0
Dimensão “Atenção à saúde da mulher”	66,4	68,1	15,7	0,0	97,9

DP: desvio padrão.

A Tabela 4 mostra a classificação dos serviços em grupos de qualidade, de acordo com a distribuição do desempenho dos serviços nos domínios, sendo o G1 o grupo de menor qualidade na organização das ações de saúde da mulher; e o G3 o grupo de maior qualidade.

**Tabela 4 Classificação:** dos serviços em grupos de qualidade segundo K-médias, por domínios da atenção à saúde da mulher. *Sistema de Avaliação e Monitoramento de Serviços de Atenção Básica* (QualiAB), Estado de São Paulo, Brasil, 2022.

Domínio	G1	G2	G3
Atenção ao planejamento reprodutivo	51,3	63,5	81,1
Atenção às mulheres em situação de violência	16,9	29,9	77,2
Prevenção e rastreamento dos cânceres de mama e útero	46,4	67,4	78,3
Atenção ao pré-natal de baixo risco	44,2	60,9	69,6
Atenção ao puerpério	40,0	85,3	93,7

G1: grupo com menor qualidade; G2: grupo com qualidade intermediária; G3: grupo com maior qualidade.

A variável “porte populacional” apresentou uma associação significativa com a qualidade dos serviços. Conforme os resultados apresentados na Tabela 5, os serviços de APS localizados em municípios com os seguintes estratos populacionais: menos de 10 mil habitantes, de 10 mil a 50 mil habitantes e de 50 mil a 200 mil habitantes, demonstraram uma redução de 0,271; 0,177 e 0,179 vezes, respectivamente, nas chances de estarem no grupo de melhor qualidade (G3), quando comparadas aos serviços de municípios com mais de 200 mil habitantes.

**Tabela 5 Modelo:** de regressão linear com resposta multinomial para explicar a qualidade das unidades. *Sistema de Avaliação e Monitoramento de Serviços de Atenção Básica* (QualiAB), Estado de São Paulo, Brasil, 2022.

Variável independente	G2			G3		
	OR	IC95%	Valor de p	OR	IC95%	Valor de p
Porte populacional do município (habitantes)						
< 10.000	0,512	0,305-0,860	0,011	0,271	0,157-0,467	< 0,001
10.000-50.000	0,475	0,315-0,716	< 0,001	0,177	0,115-0,273	< 0,001
50.000-200.000	0,594	0,401-0,880	0,009	0,270	0,179-0,408	< 0,001
> 200.000 *						
Tipo de serviço						
USF	1,169	0,582-2,350	0,661	1,106	0,493-2,484	0,807
Misto	0,741	0,374-1,467	0,390	0,557	0,250-1,239	0,151
UBS *						
Instituição que exerce gestão administrativa						
Outros	0,293	0,092-0,931	0,038	0,304	0,092-1,009	0,052
Secretaria Municipal de Saúde	0,358	0,172-0,742	0,006	0,301	0,143-0,633	0,002
Fundação ou Organização Social *						
Localização						
Rural	0,918	0,559-1,508	0,737	0,968	0,570-1,645	0,905
Urbano-periférica	0,969	0,719-1,307	0,838	0,821	0,593-1,136	0,235
Urbano-central *						
ACS na equipe						
Sim	0,803	0,385-1,667	0,560	0,936	0,389-2,249	0,882
Não *						
Captação de gestantes por ACS						
Sim	2,427	1,599-3,683	< 0,001	3,787	2,315-6,195	< 0,001
Não *						
Utilização de dados epidemiológico no planejamento						
Sim	2,790	2,136-3,644	< 0,001	8,932	6,420-12,426	< 0,001
Não *						
Periodicidade de reunião de equipe						
Semanal ou quinzenal	2,667	1,860-3,825	< 0,001	5,857	3,831-8,952	< 0,001
Mensal	1,935	1,371-2,731	< 0,001	2,966	1,948-4,516	< 0,001
Bimestral ou intervalos maiores	1,452	0,886-2,380	0,139	2,137	1,195-3,823	0,010
Não tem reunião de equipe *						

ACS: agente comunitário de saúde; G2: grupo com qualidade intermediária; G3: grupo com maior qualidade; IC95%: intervalo de 95% de confiança; OR: *odds ratio*; UBS: unidade básica de saúde; USF: unidade de saúde da família.

* Nível de referência.

Sobre tipo de gestão administrativa, os dados apresentados na Tabela 5 mostram que os serviços de administração direta municipal tiveram redução de 0,358 vezes de estar no G2 e de 0,143 vezes de estar no G3, quando comparado a serviços de gestão realizada por organização ou fundação social.

Os serviços de APS que realizam captação de gestantes por ACS apresentaram duas vezes mais chances de estar no G2 e três vezes mais chances de estar no G3, quando comparado aos que não o realizam. Esse cenário se repete quando analisamos os serviços que referem utilizar dos dados epidemiológicos no planejamento das suas ações, com cerca de duas vezes mais chances de estar no G2 e nove vezes mais chances de estar no G3, quando comparado aos que não o fazem.

Outra variável que teve associação positiva na qualidade dos serviços foi a periodicidade de realização de reuniões de equipe, mostrando que quanto mais frequentes, maior a chance de estar nos grupos de melhor qualidade, sendo que no caso das reuniões semanais ou quinzenais, a chance de estar no grupo de melhor qualidade (G3) aumenta em cerca de seis vezes, comparado aos serviços sem reuniões de equipe. As variáveis de tipo de serviço e presença de ACS na equipe não tiveram associação significativa.

## Discussão

Em que pese a atenção à saúde da mulher ser um dos segmentos mais tradicionais na agenda das políticas públicas de saúde, os resultados apresentados mostram que existe um distanciamento entre o que é proposto para a atenção à saúde da mulher na APS e o que é realizado no cotidiano dos serviços avaliados, mesmo quando se olha para indicadores relacionados ao recorte materno-infantil.

O desempenho geral de 66,4% aponta para uma implementação insatisfatória dessas ações, refletindo um reconhecimento relativamente baixo da organização de ações segundo o que é preconizado para uma atenção integral à saúde da mulher pela PNAISM e segundo protocolos nacionais adotados pelo Sistema Único de Saúde (SUS).

Sabe-se que os serviços de APS lidam com uma ampla carga de responsabilidades e atribuições, o que torna o trabalho nesse nível de atenção extremamente desafiador. Entretanto, a melhoria da qualidade da assistência à saúde da mulher, inclusive quando abordado o segmento materno-infantil, mantem-se como prioridade na agenda política das últimas décadas. Tais movimentos consideram as ações de atenção à saúde da mulher como primordiais e de competência da APS ^1^ ^,^ ^25^ ^,^ ^26^.

O domínio de atenção ao puerpério trouxe altas frequências de indicadores, mostrando a valorização das ações direcionadas a esse momento. Entretanto, observa-se um predomínio de ações voltadas à saúde da criança e contracepção, e a secundarização das relacionadas à avaliação das condições sociofamiliares e do sofrimento mental. Esses resultados corroboram o estudo de Baratieri & Natal ^27^ que traz a atenção ao puerpério na APS como uma atenção ainda fragmentada, com foco prioritário na saúde da criança, reduzindo este momento da vida da mulher ao papel de “mãe”, e não direcionado às necessidades como um todo.

Apesar da expansão da cobertura do pré-natal no Brasil, a inadequação da atenção prestada é uma problemática trazida por diversos estudos ^4^ ^,^ ^5^ ^,^ ^6^ ^,^ ^7^ e sustentada pelo desempenho apresentado nesse domínio. Cunha et al. ^7^ avaliam a atenção ao pré-natal no Brasil, mostrando baixas porcentagens de municípios com adequação do pré-natal, tanto em aspectos estruturais (32,6%) quanto operacionais (24,6%).

Dentro desse domínio, podemos observar uma maior frequência de indicadores considerados como mais “tradicionais” na atenção ao pré-natal, como a realização de testes rápidos de gravidez e convocações de gestantes faltosas, porém, quando olhamos para indicadores que representam conquistas mais recentes, como, por exemplo, a garantia da visita prévia da gestante à maternidade, vemos uma maior fragilidade em sua execução.

Observou-se uma alta frequência de serviços com respostas positivas a indicadores relacionados à prevenção da transmissão vertical da sífilis durante o pré-natal, o que representa um passo importante em busca da eliminação da sífilis congênita. Houve um aumento de 12% na frequência de serviços que realizam o tratamento com penicilina benzatina para gestante e sua parceria na própria unidade, quando comparado à avaliação realizada nos serviços da APS paulista com dados do inquérito QualiAB de 2017 ^28^.

Chama atenção que menos de 2% dos serviços relatam considerar se a gravidez é desejada ou não antes de dar o resultado do teste de gravidez. A integralidade no cuidado à mulher exige que os profissionais de saúde saiam do automatismo da simples aplicação de protocolos - sejam de início de pré-natal ou de prescrição de métodos anticoncepcionais - e identifiquem o contexto em que acontece essa possível gestação, ou seja, quanto é ou não desejada, se foi planejada, se conta com o apoio do parceiro sexual, entre outros aspectos que irão orientar os cuidados necessários a partir do resultado ^21^ ^,^ ^24^.

Em relação ao planejamento reprodutivo observou-se uma alta frequência de disponibilidade de anticoncepcionais, ainda que se espere a disponibilidade desses em 100% dos serviços. Estudo de Farias et al. ^29^ também demonstrou um maior acesso aos anticoncepcionais orais em detrimento dos injetáveis.

O acesso universal a métodos anticoncepcionais no SUS é uma das grandes conquistas dentro do campo da saúde da mulher e da saúde sexual e reprodutiva. Sua introdução no sistema de saúde é fruto de iniciativas para controle de natalidade, e ainda hoje podemos ver resquícios dessa intencionalidade nas ações de planejamento reprodutivo, ressaltada pela baixa frequência de ações educativas direcionadas a essa temática ^30^. Isso traz à tona a necessidade de olhar para a saúde da mulher a partir de outra perspectiva, o da promoção da autonomia sobre as suas escolhas reprodutivas.

Os cânceres de mama e colo de útero representam o primeiro e terceiro tipo de câncer mais frequente entre as mulheres no Brasil, respectivamente ^31^. Estratégias populacionais de rastreamento desses agravos em mulheres na faixa etária com maior risco de incidência se mostram eficazes na detecção precoce e melhora no prognóstico ^32^. Na avaliação dos serviços aqui realizada observa-se que existe uma falha na adequação dos critérios de rastreamento na população-alvo tanto para solicitação de mamografia como para coleta do exame citopatológico de colo do útero (Papanicolau), sendo muito mais expressiva no segundo.

A taxa de cobertura do Papanicolau compõe o conjunto de indicadores do Previne Brasil - modelo de financiamento federal para o custeio da APS introduzido em 2019. Estudo realizado por Schönholzer et al. ^12^ mostra que esse indicador tem atingido uma média de 14,2% no país, sendo que nenhum estado brasileiro alcançou a meta de 40% de cobertura na APS, o que seria ainda muito distante do necessário para um maior impacto na incidência do câncer de colo uterino.

A atenção às mulheres em situação de violência é o domínio com menor desempenho no conjunto de serviços avaliados, o que reflete a invisibilidade dessa temática, fomentada pela falta de capacitação dos profissionais de saúde para acolher, identificar e lidar com essas questões ^33^ ^,^ ^34^.

Claramente, podemos ver uma maior frequência de indicadores direcionados à realização de procedimentos e de prevenção de agravos, observando uma fragilidade naqueles relacionados à promoção da saúde, como as ações educativas. Tal achado chama a atenção para a necessidade de olhares mais críticos sobre o papel da APS no fortalecimento da autonomia da mulher sobre sua saúde. A educação em saúde é um processo intencional de trocas de saberes e experiências, no qual os sujeitos envolvidos possam ter domínio sobre o seu corpo mediante a aprendizagem contínua, tornando-os capazes de transformar a sua própria vida e realidade ^35^.

A variável relacionada à presença de ACS na equipe não teve resultado estatisticamente significativo na regressão multinomial, porém, os resultados mostram que a captação de gestantes realizadas por esses profissionais tem impacto na qualidade da assistência prestada, ampliando a capacidade de identificação oportuna para início precoce do pré-natal. A atuação do ACS no território favorece a longitudinalidade do cuidado, mediante a identificação das necessidades de saúde da população e maior integração com as famílias e comunidade ^36^. Os achados deste estudo corroboram com o estudo de Sanine et al. ^37^, que mostrou que conhecer o ACS da área de referência favorece o início precoce do pré-natal.

A forma de organização do processo de trabalho das equipes também revelou aspectos que impactam na qualidade dos serviços, como a periodicidade de realização das reuniões de equipe, que, conforme sejam mais frequentes, aumentam as chances de estar entre os grupos de melhor qualidade. As reuniões são espaços potentes de fortalecimento do processo de trabalho na APS, promovendo o vínculo entre os profissionais e o (re)conhecimento das necessidades sentidas no cotidiano de trabalho ^38^. Também são fundamentais para a educação permanente e para a construção de planos de intervenção conjuntos entre as equipes multiprofissionais e as equipes vinculadas ^39^.

Outra variável que mostrou influência na qualidade dos serviços foi a utilização dos dados epidemiológicos no planejamento dos serviços. O conhecimento destes dados permite a identificação dos agravos de maior ocorrência na população adscrita e o desenvolvimento de estratégias que impactem diretamente nesse cenário, produzindo, assim, melhores resultados por utilizar a análise situacional de seu território ^40^.

É importante ressaltar que este estudo utilizou dados de inquérito realizado em 2022, momento no qual o mundo ainda experienciava a pandemia causada pela COVID-19. A APS teve um papel essencial nesse cenário, se tornando responsável por ações preventivas, de vigilância e monitoramento dos casos suspeitos e diagnosticados, atendimento à demanda espontânea de sintomáticos respiratórios, entre outros. As mudanças impostas na rotina e organização desses serviços comprometeram a frequência e a qualidade de ações programáticas, como o pré-natal e a vigilância em saúde ^41^ ^,^ ^42^.

Este estudo apresenta como limitação o poder de generalização dos resultados, uma vez que retratam apenas os serviços da APS paulista que participaram da avaliação, além das limitações dadas pelo próprio recorte temporal que os estudos transversais fazem da realidade. O uso de informações extraídas de inquérito voltado para avaliação do conjunto de ações sob responsabilidade da APS implicou uma limitação de variáveis relacionadas à saúde da mulher. Porém, a avaliação da organização do processo de trabalho dos serviços no contexto de 2022 destaca pontos que exigem mudanças e medidas de planejamento e gestão, contribuindo para a melhoria da qualidade da atenção à saúde prestada.

## Conclusão

Este estudo revelou uma grande diversidade de desempenho entre os serviços de APS avaliados e permitiu identificar potencialidades e fragilidades na atenção à saúde da mulher. Ações como a disponibilização de métodos contraceptivos mais utilizados como anticoncepcionais orais e injetáveis, e preservativos; prevenção de câncer de mama em atenção a queixas ou em condições de risco diferenciado; rotinas básicas do pré-natal de baixo risco e a atenção ao puerpério revelam a realização de ações básicas, mas insuficientes, considerando o grau de prioridade dada pelas políticas de atenção à saúde da mulher e a antiguidade de grande parte dos protocolos disponíveis.

Desse modo, a avaliação apontou dados que precisam desencadear mudanças a curto e médio prazos, como em relação à inadequação dos critérios de rastreamento de câncer de colo de útero e mama; à falta de capacitação das equipes para reconhecer e acolher as demandas relativas às mulheres em situação de violência; à baixa ocorrência de ações relacionadas à saúde sexual e reprodutiva de adolescentes; e à carência de ações educativas e de promoção à saúde.

O fato de que a identificação como USF e a presença de ACS não se associarem a um melhor desempenho, contrariando o apontado por outros estudos, coloca em destaque a importância em investir em mudanças na organização do processo de trabalho. Não se questiona a maior qualificação que as USF tendem a ter em relação ao modelo tradicional, mas sim qual modelo de atenção está sendo operado, e, nesse sentido, o quanto algumas USF estão reproduzindo o modelo simplificado que se quer superar. Por isso, o trabalho do ACS se colocou como um bom indicador, pois o que qualificou a atenção à saúde da mulher foi a ação realizada e não sua simples presença como profissional da equipe.

O estudo mostrou de grande importância o fortalecimento do trabalho em equipe nas unidades de APS, para o qual necessita de espaços de reunião para que tais unidades possam incorporar ações de planejamento e medidas para pleno conhecimento e interação com a comunidade do território, assim como para aprimoramento de projetos de cuidado individual.

Esses são aspectos de grande governabilidade da gestão municipal e das equipes locais. Ainda que se deva atentar para a interdependência entre a macro e a micro gestão do trabalho, não se deve subestimar o potencial de mudanças da organização do trabalho no cotidiano dos serviços, especialmente quando as equipes conseguem desenvolver um trabalho integrado com a comunidade do território. Tanto em nível municipal como local faz-se necessário investir na qualificação dos profissionais, por meio de capacitações técnicas e de educação permanente, tanto para execução dos protocolos de maneira efetiva quanto para o desenvolvimento de olhares mais críticos sobre o papel da APS na construção da autonomia da mulher sobre sua saúde.
